# Older Adults Who Experience Their Lives to Be Completed and No Longer Worth Living: A Systematic Mini-Review Into Used Terminology, Definitions, and Interpretations

**DOI:** 10.3389/fpsyg.2021.734049

**Published:** 2021-10-21

**Authors:** Judith E. Appel, Els J. van Wijngaarden

**Affiliations:** ^1^Faculty of Psychology and Educational Sciences, Research Group School Psychology and Development in Context KU Leuven, Leuven, Belgium; ^2^Research Foundation Flanders, Brussels, Belgium; ^3^Research Group Care Ethics, University of Humanistic Studies, Utrecht, Netherlands

**Keywords:** systematic review, tiredness of life, life-weariness, completed life, older adults, euthanasia and assisted suicide, suicidal ideation, death wishes

## Abstract

In the Netherlands and in Belgium, a political debate emerged regarding the possibility of euthanasia and assisted suicide (EAS) for older adults who experience their lives as completed and no longer worth living, despite being relatively healthy. This mini-review aimed to (1) present an overview of the terms used to denote this phenomenon as well as their definitions and to (2) explore how the underlying experiences are interpreted by the study authors. A systematic search was performed in Web of Science, MEDLINE, PsycINFO, and CINAHL, yielding 35 articles meeting the selection criteria. We selected empirical, English-language articles published in peer-reviewed journals. Participants had to have a first-person experience of the phenomenon or be assessed for it, or have a third-person experience of the phenomenon. Results show that the terms tiredness of life (ToL) and weariness of life (WoL) were used most frequently, also in the broader literature on suicidal expressions across the life span. Many studies mentioned operational definitions or synonyms rather than theoretical definitions. Moreover, inside the EAS debate, the term ToL was more common, its definition incorporated death wishes, and it was regularly framed existentially. Outside of this debate, the phenomenon was generally considered as a part of suicidal ideation distinct from death wishes, and its experience was often associated with underlying psychopathology. We discuss the need to establish consensus definitions and conclude that only a multidimensional view may be suitable to capture the complex nature of the phenomenon.

## Introduction

Old age is associated with numerous losses, such as of cognitive and physical abilities and social ties. Still, the majority of older adults maintains high levels of life satisfaction up to an advanced age (Gana et al., 2013), with reductions in wellbeing usually only being observed in the very last phase of life (i.e., terminal decline; Gerstorf et al., 2008). A number of older adults, however, appear to cope less well with the challenges of late life and start to lack a sense of meaning. They develop the feeling that their life is not worth living anymore, sometimes reporting an associated death wish. These experiences also seem to develop in the absence of severe physical and mental disorders (van Wijngaarden et al., 2015; Van Humbeeck et al., 2020).

Research into this phenomenon is still an emerging area. Different terms, such as completed life, tiredness of life (ToL), or life fatigue, have been used to denote the phenomenon, but the terms lack agreed-upon definitions as well an integration with other research areas. Additionally, the terminology is influenced by the political debate that emerged in the Netherlands and in Belgium, surrounding the possibility of euthanasia and assisted suicide (EAS) for older adults experiencing their life as not worth living anymore (e.g., Oosterom and van de Wier, 2020). For example, the term completed life is a literal translation of the Dutch term “*voltooid leven*,” which is central in the EAS debate in the Netherlands. Hence, to advance the knowledge on the phenomenon, the use of consistent and well-defined terms as well as an integration with the broader literature is needed.

This mini-review therefore has two main aims: First, we seek to present an overview of the terms that have been used in the empirical literature to describe the phenomenon that older adults experience their lives as completed and no longer worth living. We will present both definitions/descriptions of these terms and the contexts and populations they are associated with, thus not restricting our search to their use in late life populations. Second, we aim to explore how the underlying experiences are interpreted by study authors (e.g., as a normative experience, a pathological condition, an existential problem).

By taking a broad approach, we hope to gain insight into the extent to which the various terms describe the same or differing (severities of) underlying phenomena, in how far they reflect a different theoretical approach, and where terminological and theoretical overlap with other research areas might lie.

## Materials and Methods

### Search Strategy

We systematically searched four databases: MEDLINE, PsycINFO (both *via* Ovid), CINAHL (*via* EBSCO), and Web of Science core collections. Databases were searched from inception until 16-03-2021.^1^ Keywords were completed life, tiredness of life, suffering from life, weariness of life, finished with life, life fatigue, and their variations (e.g., “tired of living”; based on van Wijngaarden, 2016; for the full search strings per database see OSF). In addition to the database search, we examined the reference lists of included articles as well as of related reviews that were identified through our search for further references.

### Study Selection and Data Extraction

Articles were eligible if they (a) were written in English, (b) published in a peer-reviewed journal, (c) discussed the topic of “tiredness of life” (or an alternative term), (d) used an empirical study design, and (e) investigated either participants with a personal, first-person experience of the phenomenon or assessed them for this experience, or studied participants with a third-person experience of the phenomenon (such as caregivers). We focused on empirical English-language articles and a study population with experience with the phenomenon as we were interested in the international use of the terminology compared to the use within the national political debate.

Study selection was performed using Mendeley and Rayyan (Ouzzani et al., 2016). In the title/abstract screening, the first 50 references were screened independently by both authors, reaching an agreement of 98%. Consequently, the first author conducted the rest of the title/abstract screening as well as the full-text screening and data extraction. From all studies, we extracted general study details (first author, year of publication, country, and study design), study focus (main aims and results), terminology, definition/description/operationalization, and interpretation/framing. Depending on the type of sample studied, we also extracted information regarding participants’ age and gender (first-person experience) or role/occupation and work experience (third-person experience). Doubts during screening and extraction were discussed with the second author.

### Data Analysis

For the first aim, we made an overview of all (main) terms used for the phenomenon in the articles and then grouped them (e.g., combining tired of living and tiredness of life). For each of these terms, we then created an overview of the characteristics of the studies that applied a given term – namely country, publication year range, age range, type of sample, first- versus third-person perspective, and conducted inside versus outside EAS debate – as well as the definitions given. For the second aim, we assigned one or multiple primary and secondary perspectives to the articles based on the authors’ explicit descriptions and interpretations as well as related phenomena and mentioned theories. We tried to stay as close as possible to the authors’ wording. Then, similar to the first aim, we made an overview of study characteristics for every perspective.

## Results

### Study Selection and Characteristics

The database search identified 533 records. After duplicate deletion, 299 records remained. Sixty full-texts were assessed for eligibility, leading to 35 studies being included in the synthesis. The reference list search did not identify any additional articles (for PRISMA flow chart see Supplementary Figure S1; Moher et al., 2009). An overview of the included articles is presented in Tables 1 and 2. Studies were published between 1972 [9] and 2021 [10;35] and conducted in 14 different countries. Twenty-seven studies included participants that reported a first-person experience of the phenomenon or were assessed for its presence [Table 1 (1–27)], whereas eight studies investigated participants with a third-person experience of the phenomenon (Table 2 [28–35]). All studies on third-person experiences were conducted in the Netherlands or in Belgium and were related to the political debate surrounding EAS. Regarding the 27 studies on participants with a first-person experience, the age of samples studied varied from young people to oldest old individuals. Overall, the majority of studies focused on older adults [1–2;10–11;14;22–23;25;27], the general population [4;6–7;15–19], or healthcare professionals [28–35]. Other samples investigated were individuals who died by suicide, young adults, individuals who requested EAS, and medical samples. Most studies took a quantitative approach [1–9;11–23;25–26;28–31;33;35]. The most prevalent type of research question concerned the prevalence and/or predictors of the ToL phenomenon [1;2;4;6–8;11–13;15–19;21–22;25], while others examined ToL as a predictor, aimed at gaining an in-depth understanding of ToL or related phenomena, or concerned end-of-life decision making.

**Table 1:** Characteristics of studies on first-person experience of the phenomenon.

Study details	Main aims	Main results	Participants details	Main term	Definition	Framing
[1] Barnow and Linden, 1997, Germany, Quantitative	Investigate prevalence of suicidal ideation in very old persons.	Weighted 14.7% of the elderly population experienced tiredness of life (ToL), with more females than males.	*N*=516*Type*: late life, stratified sample*Age*: R=70–105*Gender*: n.a.	Tiredness of life	Part of suicidal ideation; “feels life not worth living”	Psycho-pathological
	Investigate whether suicidal expressions show (similar/different) trends over time and gender.	Significant interaction between age groups and severity of suicidality, but increase especially marked in the more severe categories.				
			[2] Barnow and Linden, 2000, Germany, Quantitative				Investigate whether the wish to die or suicidal ideation is widespread among the elderly, and whether it is related to psychiatric morbidity.	The higher the intensity of the suicidality, the higher the probability that the person is suffering from a psychiatric disorder.	*N*=54 (from study [1])	Tiredness of life	“feels life not worth living”	Psycho-pathological (rational)
									*Type*: late life, “suicidal group”			
*Age*: M(SD)=89.58 (8)												
*Gender*: 61.1% female												
[3] Brådvik and Berglund, 1993, Sweden, Quantitative	Compare characteristics of individuals with melancholia who died by suicide to those with melancholia.	Weariness of life (WoL) in connection with depression was more frequently reported by people who died by suicide who had brittle/sensitive personality. Even within brittle/sensitive group, WoL was more frequent among people who died by suicide than controls.	*N*=89 (per group)	Life-weariness	Part of suicidal behavior	Psycho-pathological
			*Type*: case records of people who died by suicide, matched controls			
			*Age*: M_male_=45; M_female_=48			
			*Gender*: 51 women			
[4] Brunoni et al., 2015, Brazil, Quantitative	Investigate suicidal ideation and its association with clinical and demographic variables (in Brazil).	3.11% of participants presented WoL thoughts in the previous 7days.Common mental disorder (CMD) and WoL thoughts were robustly associated.Other positive associations: female, single, past stressful life event, antidepressant use, poor perceived health; negative associations: Catholic/Espiritism religion, college education.	*N*=15.105	Life-weariness thoughts	Part of suicidal ideation; ToL or tedium vitae; “feels life not worth living”	Psycho-pathological (societal)
			*Type*: civil servants			
			*Age*: R=35–74 (22% 35–44; 39% 45–54; 28% 55–64; 11% 65–74)			
		*Gender*: 54% female				
			[5] Copeland, 1987, United States, Quantitative				Analyze case files and suicide notes of people who died by suicide due to drowning.	Regarding reasons for suicide, depression is common and often concerns poor health, the death of a loved one, or being “tired of living” (ToL mentioned by 4.3%).	*N*=70	Tired of living	n.a. (but experience self-reported in suicide notes)	Psycho-pathological
									*Type*: case files of people who died by suicide			
		*Age*: 1.43% 0–20; 4.3% 21–25; 11.4% 26–30; 4.3% 31–35; 4.3% 36–40; 10% 41–45; 7.1% 46–50; 4.3% 51–55; 2.9% 56–60; 5.7% 61–65; 5.7% 66–70; 38.6% 70+										
*Gender*: 41.4% female												
[6] Dennis et al., 2007, United Kingdom, Quantitative	Examine association between mental disorder and spectrum of suicidal ideation. Explore how social and general health factors correlate with suicidal ideation in ‘young elderly’ and compare it to younger age groups.	Past year ToL was reported by 6%, with younger people being more likely to experience it.	*N*=8,580	Tiredness of life	Part of suicidal ideation; “thought life not worth living”	Psycho-pathological
			*Type*: nationally representative sample			
		CMD and depression strongly associated with ToL (stronger in 55–74 age group compared to younger people). For age 55–74 the strongest relationships for ToL were: poor perceived social support, widowhood, fair/poor self-rated general health, limitations in activity of daily living.	Age: R=16–74 (28.9% 16–34; 39.5% 35–54; 31.6% 55–74)				*Gender*: 55.1% female
	[7] Hällström, 1977, Sweden, Quantitative		Show the frequency of various degrees of suicidal tendency earlier in life in a representative group of middle-aged women.				22.3% of participants had the feeling that life was not worth living.	*N*=800	Life-weariness	Part of suicidal ideation; “felt life not worth living”	Psycho-pathological
*Type*: representative group, middle-aged											
*Age*: R=38–54											
*Gender*: 100% female											
[8] Herrera Rodríguez et al., 2006, Nicaragua, Quantitative	Examine the prevalence of self-reported suicidal expressions among young people in Nicaragua, identify associated socio-demographic factors.	33.5% reported last year WoL.	*N*=278	Life-weariness	Part of suicidal behavior; “felt life not worth living”	Psycho-pathological
		There were no significant associations in either gender to socio-demographic conditions.	*Type*: young people, randomly selected			
			*Age*: R=15–24, M_male_=19.6, M_female_=20.0			
			*Gender*: 47.8% female			
[9] Humphrey et al., 1972, United States, Quantitative	Systematically investigate certain problematic life events that precede suicide.	ToL was generally experienced after problems with drinking/family/work/sex and prior psychiatric treatment, and preceded suicide attempts and threats.	*N*=158	Tiredness with life	“despondency with life” (used interchangeably)	Psycho- pathological (social)
			*Type*: patient charts, people who died by suicide			
			*Age*: n.a.			
			*Gender*: n.a.			
[10] Koskinen et al., 2021, Finland, Qualitative	Deepen understanding of older men’s life after the loss of their life partners.	Informants experienced loneliness, which could make life feel empty and lead to thoughts that living is no longer worthwhile.	*N*=5	Life weariness	n.a (but experience emerged in interviews)	Existential
			*Type*: late life, lost life partners			
			*Age*: R=81–91			
			*Gender*: 0% female			
[11] Lindner et al., 2014, Germany, Quantitative	Gain more insight into the inner world, intrapsychic conflicts and psychosocial conditions of suicidal geriatric patients.	Patient group: trend of more experiences with death of an important person, bombing, sexual abuse, displacement/flight, persecution; more depression; more psychiatric/psychotherapeutic treatments. Triggers of lifetime suicidal ideation differed from triggers of current suicidal ideation.	*N*=20 (per group)	Life weariness	Part of suicidal ideation; “thought life not worth living”	Psycho- pathological
			*Type*: late life; patients (suicidal or T/WoL), matched controls			
			*Age*: M(SD)_patient_=79.3 (7.6), M(SD)_control_=81 (5.9)			
			*Gender*: 65/70% female (patient/ control)			
[12] Ojagbemi and Bello, 2019, Nigeria, Quantitative	Investigate the association between first-ever stroke and tedium vitae using a comparative cross-sectional design.	Tedium vitae was experienced by 12.3% patients compared with 3.8% controls. Retired patients were more likely to report tedium vitae.	*N*=130 (per group)	Tedium vitae	Tedium vitae represents the thought that life is not worth living or feeling tired of life; “felt life not worth living”	Psycho- pathological
			*Type*: stroke survivors, matched controls			
			*Age*: M(SD)_patient_=59.5 (11.1), M(SD)_control_=58.8 (11.2)			
			*Gender*: 53.8/51.5% female (patient/control)			
[13] Omma et al., 2013, Sweden, Quantitative	Explore suicidal expressions among young Sami compared to Swedes in general.	Suicidal expressions were common among young Sami and Swedes but the Sami reported a higher prevalence of WoL (63% *vs*. 50%), death wishes and suicidal ideation. Per group, women reported more WoL and death wishes than men.	*N*=516; 218	Life weariness	Part of suicidal expressions; “felt life not worth living”	Existential, cultural (psycho- logical)
			*Type*: young Swedish Sami; age-matched Swedes as reference			
			*Age*: R=18–29			
			*Gender*: 57.2/51.8% female (Sami/reference)			
[14] Pac et al., 2013, Poland, Quantitative	Assess change in the role of gender-related mortality predictors over 20years in older Krakow citizens.	A strong feeling of WoL was an important all-cause mortality risk factor for women (but not for men). It was related to the 14% increase in mortality.	*N*=2,472	Feeling of life-weariness	n.a.	n.a.
			*Type*: late life, random sample 65+			
			*Age*: M(SD) =72.4 (5.7)			
			*Gender*: 65.1% female			
[15] Ramberg and Wasserman, 2000, Sweden, Quantitative	Investigate differences in prevalence of suicidal thoughts and suicide attempts between mental health-care staff (and professional categories) and general population.	Taking age and gender into account, disparities in lifetime thoughts of life is not worth living and death wishes between professional categories are significant, with fewer nurses than psychologists/social workers having had thoughts of life not worth living and death wishes.	*N*=1,010; 8,171	Life-weariness	Part of suicidal behavior; “felt life not worth living”	Psycho- pathological
			*Type*: mental health-care staff; general population			
			*Age*: M_staff_=41.6 (R=19–63), M_general_=42.7 (R=20–66)			
			*Gender*: 71.8/53.4% female (staff/general)			
[16] Rancāns et al., 2003, Latvia, Quantitative	Assess prevalence of suicidal behaviours in the general population, identify risk groups, examine the suggested continuous sequence of suicidal behaviours.	19.7% (weighted 36.3%) of the population reported WoL.	*N*=667*Type*: general population*Age*: 28% 18–34; 33.1% 35–54; 38.9% 55+*Gender:* 71.8% female	Life-weariness	Part of suicidal behaviors; “felt life not worth living”	Psycho- pathological
		Risk factors: non-cohabitation status, lower education (males), higher education (females).				
		39.1% reported non-continuous pattern of past year suicidal behaviours (13.4% when starting from death wishes).				
			[17] Rancāns et al., 2016, Latvia, Quantitative				Determine last year prevalence of different types of self-reported suicidal behaviour, socio-demographic risk factors, sequence of suicidal behaviours.	Last year prevalence for WoL was 16.6%, with a significantly higher prevalence for women.	*N*=2,816*Type:* representative sample 18+, weighted*Age*: weighted 42.3% 15–34; 41.2% 35–54; 16.4% 55–64	Life-weariness	Part of suicidal behavior; “felt life not worth living”	Psycho- pathological
Risk factors (mild types, both genders): older/middle age, non-cohabitation status, low education.												
79.6% reported continuous pattern of past year suicidal behaviours.												
	*Gender*: weighted 51.4% female											
[18] Renberg, 2001, Sweden, Quantitative	Assess prevalence and incidence of suicidal expressions in a general population, make comparisons over time, identify risk groups, examine continuous sequence of suicidal expression.	In 1986 (survey 1), higher combined prevalence for all types of suicidal expression for: women, younger groups, persons living alone. In 1996 (survey 2), higher prevalence for persons living alone.	*N*=509/623*Type*: general population; survey 1/2*Age*: Survey 1= 31% 18–30; 23% 31–40; 21% 41–50; 18% 51–60; 7% 61–65; Survey 2=29% 18–30; 20% 31–40; 24% 41–50; 20% 51–60; 7% 61–65	Life-weariness	Part of suicidal process; “felt life not worth living”	Psycho- pathological
		No simple cumulative relationship between the different types of suicidal expression.				
			*Gender*: 56/55% female (survey 1/survey 2)			
			[19] Spiers et al., 2014, United Kingdom, Quantitative				Test the hypothesis that age-specific prevalence of suicidal ideation declined between 2000 and 2007, in line with the incidence of suicide.	Little evidence of trends in prevalence of suicidal ideation.Prevalence of suicidal ideation in the past year followed a W-shaped profile with age, with peaks at the transition to adulthood, in the forties, and in the oldest participants.	*N*=6,799/6,815*Type*: representative sample; survey 1 (2000)/survey 2 (2007)*Age*: R_1_=16–71; R_2_ = 16–78*Gender*: n.a.	Tiredness of life	Part of suicidal ideation; “felt life not worth living”	Psycho- pathological
[20] Stanford et al., 2017, Australia, Quantitative	Identify factors that predict self-harm.Assess effect of self-harm on subsequent outcomes.	Without previous self-harm: ToL univariately strongest predictor of self-harm (non-significant when controlled for depression, abuse, stress). With previous self-harm: ToL, stress, and number of dieting behaviors predictors of self-harm.		*N*=5,765*Type*: young women, random, survey s1–s5*Age*: R_s1_=18–23, R_s2_=22–27, R_s3_= 25–30, R_s4_=28–33, R_s5_ = 31–36*Gender*: 100% female	Tiredness of life	Part of suicidal behavior; “felt life not worth living”		Psycho- pathological (psycho- social)				
	[21] Sumathipala et al., 2004, Sri-Lanka, Quantitative			Examine whether patients with suicidal thoughts and WoL volunteer them.					No patient volunteered suicidal ideation or WoL.When directly questioned, 59% in index group and 26% of controls admitted experiencing WoL.In both groups, people with WoL, hopelessness, and suicidal ideations had a higher probability of CMD.				*N*=100 (per group)*Type*: index group (medically unexplained multiple symptoms), random control patients*Age*: R_control_ = 16–65 (index group n.a.)*Gender:* n.a.	Life weariness	Feeling sick of life	Psycho- pathological
				Examine relationship between WoL, suicidal ideation, and underlying CMD.												
			[22] Tuvesson et al., 2018, Sweden, Quantitative				Investigate point prevalence of WoL and suicidal thoughts and their possible relationships with socio-demographic characteristics in a population of older adults in Sweden.		10.7% of respondents felt WoL.Compared to those with no WoL or suicidal thoughts, those with WoL were: older, in (semi-)urban areas, in residential care facilities/other-than ordinary homes, with low education, widowed, unmarried, divorced, born in non-Nordic European countries, with low financial resources.	*N*=7,913*Type*: late life, from 10 age cohorts (60–96+)*Age*: M(SD) = 73.2 (10.7)*Gender:* 58.9% female	Life weariness	Part of suicidal expressions	Psycho- pathological			
[23] Zawisza et al., 2015, Poland, Quantitative		Assess relationship of sleep duration and all-cause mortality among Polish community dwelling older citizens during 22years follow-up.			Those who slept fewer hours more often reported feelings of WoL.Individuals reporting no WoL had about 10% decreased risk of death as compared to the group reporting a low level of WoL.The expected U-shaped mortality risk associated with sleep duration was observed among individuals with high WoL, whereas among those without WoL the relation was linear.	*N*=2,449*Type*: late life, random sample 65+*Age*: M(SD)_male_ = 72 (5.8), M(SD)_female_ = 72.5 (5.7)*Gender*: 65% female		(Feeling of) life weariness	Long lasting psychosocial condition considering several experiences like general life dissatisfaction, long-lasting physical and mental tiredness, loss of energy and general lack of ‘internal drive’ regardless of its background (economical, psychosocial or medical)	Psycho- pathological, psycho- social, physio- logical, physical						
	Investigate modification effect of demographic, psychosocial, health- related conditions.												
		[24] Dees et al., 2011, the Netherlands, Qualitative		Explore the constituent elements of suffering of patients who explicitly request EAS, better understand unbearable suffering from the patients’ perspective.	Suffering was constituted by medical, psycho- emotional, socio-environmental, existential aspects.	*N*=31*Type*: patients who had requested EAS*Age*: R = 32–94, M = 67.9*Gender*: 54.8% female								Tired of life	n.a (but experience emerged in interviews)	Existential
			Regarding the existential dimension: hopelessness inevitably gave rise to feelings of pointlessness that resulted in ToL (54.8% mentioned ToL).													
[25] Hartog et al., 2020, the Netherlands, Quantitative	Investigate prevalence of older adults with a persistent death wish without severe illness and their characteristics, existential issues and the nature of their death wishes.				1.25% of participants reported a persistent death wish without severe illness. There was no significant overall difference in age distribution. The group with a persistent death wish had significantly worse health.	*N*=21.294*Type*: late life, representative sample Dutch adults 55+*Age*: Median_Q1–Q3_ = 65*Gender*: 50.3% female	Completed life (CL); Tiredness of life	CL: persons, mostly of old age, who do not see a future for themselves and, as a result, have developed a persistent, active death wish, without suffering that (mainly) originates in a medically classifiable condition.ToL: suffering caused by the prospect of having to continue living with a very poor quality of life, not predominantly caused by a physical or psychiatric disease, and closely associated with a death wish	Physical, social, existential							
						[26] Snijdewind et al., 2015, the Netherlands, Quantitative				Study how often the different possible outcomes of applications for EAS occur and which factors are associated with the outcome.	27.5% of requests from patients who were ToL were granted.	*N*=645*Type*: application forms, requested EAS*Age*: 10.2% <40; 21.9% 40–60; 29.1% 60–80; 38.8% >80*Gender*: 61.9% female	Tired of living				n.a (but experience self- reported in application files)	n.a.
		[27] van Wijngaarden et al., 2015, the Netherlands, Qualitative	Develop an in-depth understanding of the phenomenon that ‘life is completed and no longer worth living’.	Essential meaning of phenomenon: ‘a tangle of inability and unwillingness to connect to one’s actual life’. Constituents: loneliness; not mattering; inability to express oneself; multidimensional tiredness; aversion towards feared dependence.				*N*=25*Type*: late life, considered their lives completed*Age*: M=82*Gender*: 56% female				Completed life		n.a (but interviews conducted to describe phenomenon)	Societal, narrative, existential			

*Studies highlighted in gray are conducted outside the EAS debate. Framing in brackets refers to secondary perspectives. Study [1] and [2] as well as [6] and [19] are partially based on the same data. R. range; M(SD), mean (standard deviation); and n.a. not available*.

**Table 2:** Characteristics of studies on third-person experience of the phenomenon.

Study details	Aims	Main results	Participant details	Main term	Definition	Framing
[28] Bergman et al., 2020, the Netherlands, Quantitative	Determine frequency of consultations that are perceived as difficult by SCEN physicians, which complexities they perceive, characteristics associated with perceiving a consultation as difficult.	1 out of 5 consultations are perceived as difficult.8.4% of the consultations perceived as difficult due to ToL, while 2.7% of the consultations perceived not to be difficult concerned requests due to ToL.	*N*=498/573	Tired of living	n.a.	n.a.
			*Type:* SCEN physicians; 2015 and 2016/17 survey			
			*Work exp.:* Number consultations/year: 6.3% <5; 26.2% 5–9; 27.45% 10–14; 40.05% 15+			
[29] Bolt et al., 2015, the Netherlands, Quantitative	Describe whether physicians can conceive of granting (or have granted) EAS in patients with cancer, another physical disease, psychiatric disease, dementia or who are ToL (without severe disease).	For patients being ToL with medical grounds for suffering: 3% performed EAS, 27% finds it conceivable or has performed, 73% finds it inconceivable.	*N*=1,456 (708, 287, 461)*Type:* physicians (GPs, elderly care physicians, clinical specialists)*Work exp*. (years): R=1–42, M(SD)=18 (9)	Tired of living	Suffering caused by the prospect of having to continue living with a very poor quality of life, not predominantly caused by a physical or psychiatric disease, leading to a persistent death wish	Psycho-social, existential
		Without medical grounds for suffering: 2/18/82%				
			[30] Brinkman-Stoppelenburg et al., 2014, the Netherlands, Quantitative				Study why requests are sometimes judged not to meet requirements of due care, find out which patient/SCEN physician characteristics are associated with judgments.	The reason to request euthanasia was “being tired with life” in 6% of the cases; being ToL was associated with a higher likelihood that the requirements of due care were judged not to be met.	*N*=415	Being tired with life	n.a.	n.a.
*Type*: SCEN physicians (77% GPs, 11% nursing home physicians, 12% medical specialists); *Work exp*. (years): as SCEN, 21% <4; 55% 4–8; 24% 8+												
[31] Rietjens et al., 2009, the Netherlands, Quantitative	Study whether there are any differences between physicians, consultants and members of the review committees (RTE) in their judgements of patients’ suffering.	For ToL, 92% GPs, 97% consultants, 71% RTE could imagine such a case in their practice.	*N*=231 (115, 99, 17)*Type*: total (GPs, consultants, RTE)*Work exp*. (years): GPs=25% <10, 26% 10–20, 49% >20; Consultants =M(SD)=4.5(2.1); RTE=M(SD)=5.4 (2.7)	Tired of living	n.a.	Psycho-social, existential
		Suffering in early dementia and being ToL was least often considered unbearable (all <35%).				
			[32] Rurup et al., 2005a, the Netherlands, Qualitative				Estimate incidence of requests for EAS in the absence of a severe disease, get insight in characteristics and reasons of patients who make such requests, learn more about how physicians deal with requests.	Requests based on WoL were almost never granted.	*N*=410 (77, 125, 208)*Type*: physicians (nursing home physicians, GPs, clinical specialists)*Work exp*.: min. 2years	Weary of life	n.a. (but requests for EAS in the absence of disease are explained)	Physical, social/societal (rational)
79% had non-severe illness(es.).												
Reasons for requests: through with life (55%), physical decline (55%), ToL (48%), suffering from life (28%).												
[33] Rurup et al., 2005b, the Netherlands, Quantitative	Study to what extent being ToL, as reason to request EAS, occurs in the presence or absence of a severe disease, as well as characteristics, symptoms, and reasons of these patients, and physician decisions.	Of patients for whom ‘being ToL’ played a major role in the request, 47% had cancer, 25% had another severe disease, 28% had no severe disease.	*N*=3,994*Type*: GPs*Work exp*.: n.a.	Tired of living	n.a.	Medical
		54% of requests by patients without severe disease were refused.				
			[34] Van Humbeeck et al., 2020, Belgium, Qualitative				Acquire a deeper understanding of what it is to be a nurse in a home care context or nursing home taking care of residents/patients being ToL, and gain insight into nurses’ (a) perceptions of (b) attitude(s) toward, and (c) ways of dealing with ToL.	The confrontation with persons having ToL instigates a cognitive process of searching to understand the state a person is in, which on its turn ensues in an emotional balancing between courage and powerlessness and a behavioral approach of action or dialogue.	*N*=25	Tiredness of life	ToL (WoL, life fatigue): suffering caused by the prospect of having to continue living with a very poor quality of life, not predominantly caused by a physical or psychiatric disease, and closely associated with a death wish. This term concerns the idea that “life is not worth living, or that you’d be better off dead.”	Existential (psycho-logical)
*Type*: nurses												
*Work exp. (*years): R=1–33, M(SD)=13 (8.7), all experience with caring for patients who were ToL												
[35] Van Humbeeck et al., 2021, Belgium, Quantitative	Explore the legal understanding and attitudes of nurses and physicians regarding euthanasia within the context of ToL in older people.	Acute care healthcare professionals were more often confronted with patients who report ToL, compared to chronic care.	*N*=345 (135, 59, 75, 76)*Type*: total (GPs, geriatricians, nurses acute care, nurses chronic care)*Work exp*. (years): GPs =21% <10; 9% 10–20; 70% >20; Geriatricians=31% <10; 32% 10–20; 37% >20; Nurses acute=35% <10; 29% 10–20; 36% >20; Nurses chronic=29% <10; 29% 10–20; 42% >20	Tiredness of life	ToL (‘WoL’, ‘life fatigue’): suffering caused by the prospect of having to continue living with a very poor quality of life, not predominantly caused by a physical or psychiatric disease, and closely associated with a wish to die.	Narrative (existential)
		In case of ToL without underlying pathology, nurses showed more comprehension for the euthanasia request compared to physicians				

*All studies were conducted inside the EAS debate. Framing in brackets refers to secondary perspectives. Work exp., work experience; R, range; M(SD), mean (standard deviation); and n.a., not available*.

### Which Terms Are Used in the Literature and How Are They Defined?

Variations of the terms tiredness of life (ToL; e.g., tired of living/life) and weariness of life (WoL; e.g., life-weariness; feeling of life-weariness) were used most frequently and sometimes interchangeably. Both terms were employed in various countries, with ToL being more prevalent in the Netherlands and in Belgium and WoL being more prevalent in Scandinavian and non-western countries. Similarly, both terms have been used since the 1970s [7;9] and were still present in the recent literature [10;35]. The populations in which the terms were applied were varied and largely overlapping. However, ToL was much more common in samples of healthcare professionals and of individuals requesting EAS, or, more broadly speaking, in studies conducted within the EAS debate. Another term used as a main term in two articles, both of which were conducted in the Netherlands and related to the EAS debate was “completed life” [25;27]. Other literally translated terms from the EAS debate were hardly adopted in the empirical literature [but see 24], and never used as main terms. We did, however, find some terminological overlap in a study that interpreted WoL in the light of a theory of caring sciences that proposes the existence of different kinds of suffering, one of which is termed “life suffering”/“suffering of life” [10]. Despite not specifying this term in our keywords, another synonym for ToL/WoL was “tedium vitae.” Tedium vitae was the major term in Ojabemi et al. [12] and a synonym in Brunoni et al. [4], both studies conducted outside of the EAS debate.

In line with the previous finding, also the definitions/descriptions of all employed terms revealed much similarity. In general, many studies mentioned synonyms or operational definitions or lacked any description at all, instead of providing a theoretical definition. Again, differences primarily emerged between studies that were conducted inside versus outside of the EAS debate. Outside of the EAS debate, most studies mentioned “the thought/feeling that life is not worth living” as their (theoretical) definition/description or operationalization/measurement of the phenomenon [1;2;4;6–8;11–13;15–20]. Moreover, the majority of these studies regarded the phenomenon as a part of suicidal ideation with a low level of intent, independent of the specific term applied [1;3–4;6–8;11;13;15–20;22]. Interestingly, although these studies usually regarded death wishes as a more severe form of suicidal ideation in the suicidal process, some studies grouped ToL/WoL together with death wishes in their measurement or analysis [8;15–17;22]. Likewise, the supposed incremental relationship from ToL/WoL over death wishes to suicidal thoughts and attempts was found by some [1;17] but not all studies [16;18].

In studies conducted within the EAS debate, in contrast, the phenomenon was not explicitly linked to suicidal ideation. Multiple studies, sometimes considering WoL and life fatigue as synonyms, defined ToL as “suffering caused by the prospect of having to continue living with a very poor quality of life, not predominantly caused by a physical or psychiatric disease, and closely associated with (/leading to) a death wish” [25,29,34–35]. One study additionally provided a definition of “completed life,” namely, “persons, mostly of old age, who do not see a future for themselves and, as a result, have developed a persistent, active death wish, without suffering that (mainly) originates in a medically classifiable condition” [25]. Usually, studies employing these definitions also mentioned the experience of feeling that “life is not worth living” in their further description of the phenomenon [25,27,34–35]. Hence, definitions/descriptions provided by studies inside and outside the EAS debate partly overlapped. Within this debate, however, definitions of the phenomenon explicitly incorporated death wishes, though their exact role and severity remained unclear. Additionally, these definitions often specified the cause for the experience as at least partly situated outside of the medical domain.

### How Is the Phenomenon Interpreted?

The majority of study authors adopted a *psychopathological* perspective [1–9,11–12,15–23], thus, for instance, viewing the phenomenon as a condition closely associated with psychiatric disorders and as requiring treatment. This perspective in many cases was the only interpretation given and was taken across countries and populations. Studies from the Netherlands and Belgium and concerning healthcare professionals or individuals requesting EAS formed an exception. Indeed, all studies adopting a psychopathological perspective were conducted outside of the EAS debate. The second most common way of framing the phenomenon was *existential* [10,13,24–25,27,29;31;34–35]. These studies related the phenomenon to a lack of meaning, experiences of emptiness, or difficulties with constructing a (cultural) identity. The existential perspective was predominantly found in studies from the Netherlands and from Belgium and thus within the EAS debate, though also two Scandinavian studies on young and old adults, respectively, adopted this perspective. Notably, the psychopathological and existential perspective never appeared in combination. Merely two studies with an existential perspective additionally discussed the phenomenon’s possible relation to *psychological* (but not necessarily psychopathological) factors [13,34].

In addition, various studies interpreted the phenomenon as grounded in *social/societal/cultural* issues [4,9,13,25,27;32] or *psychosocial* problems [20,23,29,31]. These perspectives were always mentioned alongside other perspectives and were adopted independent of country and population, both inside and outside the EAS debate. Some ways of framing the phenomenon were exclusively found in studies linked to old age. These studies, for instance, stressed the importance of *physical/medical* aspects [23,25,32–33], such as of fatigue and physical deterioration, or suggested an intertwinement with *physiological* aspects [23]. Moreover, a *narrative* perspective explaining the phenomenon as a potential consequence of perceiving one’s life story as completed was adopted by two studies within the EAS debate [27,35]. Lastly, the hypothesis that life-weariness and death wishes are the result of *rationally considering* one’s life situation was put forward as a (secondary) perspective [2,32]. Importantly, other studies explicitly questioned this interpretation [25,27].

Of note, we could not infer a perspective for four studies [14,26,28,30], mainly from inside the EAS debate and concerning end-of-life decision making. Therefore, these studies were not included in this part of the analysis.

## Discussion

Some of the terms used to denote the phenomenon that relatively healthy older adults experience their life as completed and not worth living are also employed in the broader empirical literature, especially on suicidal expressions across the life span. ToL and WoL are found most frequently, with many studies mentioning operational definitions or synonyms rather than theoretical definitions. Moreover, which terms are used, how these terms are defined, and how the experience is interpreted differs depending on country and whether a study is related to the EAS debate. Inside the EAS debate, the term ToL is common, its definition incorporates death wishes, and it is regularly framed as an existential problem. Outside of this debate, the term WoL is applied as well, the phenomenon is generally considered as a part of suicidal ideation distinct from death wishes, and its experience is often associated with psychopathology.

These differences can be understood in the light of the criteria that have to be met to be eligible for EAS in the Netherlands and in Belgium, which specify that an individual’s suffering must be grounded in a medical condition (De Jong and van Dijk, 2017). There is debate about whether older adults who do not suffer from a severe somatic or psychiatric disease but experience their lives to be completed and wish for death should have the option to request EAS (van Wijngaarden et al., 2017; Holzman, 2021). The definition of ToL inside the EAS debate is thus tailored towards this very specific group and is inherently inconsistent with a strictly psychopathological framing.

Despite their differences, the two lines of research on the phenomenon have similar limitations that future research needs to address. For instance, an overall lack of theoretical definitions and an inconsistency concerning the relationship between ToL/WoL and death wishes was observed. Clarifying this question and establishing consensus definition(s) is not only crucial for comparability among studies but will simultaneously elucidate the appropriateness of viewing the phenomenon as a part of suicidal ideation. For example, while in suicide research advances have been made by distinguishing between suicidal ideation and suicide attempts (Klonsky et al., 2018), it is – especially for older adults – still unclear whether there are also qualitative differences within suicidal ideation (O’Riley et al., 2014; Van Orden and Conwell, 2016). Currently, age differences are difficult to determine, as inside the EAS debate the phenomenon is viewed as unique to late life, whereas outside this debate similar definitions are applied for varying age groups. It is conceivable, however, that reflecting about the worth of one’s life and thinking about or even hoping for one’s death can have different origins and meanings depending on an individual’s life stage. In later life, it might be indicative both of normative developmental processes of dealing with mortality as well as of underlying suicidality (Van Orden and Conwell, 2016). One possibility to gain insight into these nuances is the adoption of more diverse study designs and of theory-derived research questions that move beyond investigations of prevalence, predictors, and end-of-life decisions. Indeed, the latter has been previously emphasized with regard to late-life suicide (Van Orden and Conwell, 2016).

At the same time, combining aspects from the two lines of research could deepen the current understanding of the phenomenon. For example, the idea that “life is (not) worth living” which was included in many descriptions of the phenomenon has also been conceptualized as an evaluative component of experienced meaning in life (Martela and Steger, 2016). Thus, incorporating an existential perspective more broadly outside the EAS debate seems warranted and might offer one theoretical route forward. Concurrently, even in the study on “completed life” that selected individuals without severe diseases, individuals with death wishes displayed poor mental health and sometimes reported the lifelong presence of their death wishes (Hartog et al., 2020). Therefore, also when studying seemingly healthy samples as often done inside the EAS debate, attention for (sub-clinical) psychopathology should be kept. Actually, the distinction between existential and psychopathological factors itself might be artificial given various accounts stressing their interplay (Yalom, 1980; Maxfield et al., 2014). Similarly, results point to social-relational as well as societal-cultural influences on the experience of the phenomenon. Introducing studies on the perspectives of caregivers and relatives also outside of the EAS debate could be one way to gain more insight into these dynamics.

The current findings are strengthened by the reviews’ broad approach, including articles independent of, for example, their publication year, studied age group, or geographical location. Thereby, a comprehensive overview of research into the phenomenon was created which allows to discern variations as well as overlap in results depending on cultural developments and type of sample. At the same time, a limitation that should be kept in mind is that the findings on terminology are restricted by our keywords. There might be even more terms used to refer to the phenomenon, or related culture-specific phenomena.

Concluding, it is likely that only an integration of various perspectives will be able to do justice to the multidimensional nature of the phenomenon and can uncover potential variations in its experience across cultures, developmental stages, and health statuses. Ideally, this multidimensionality will also be reflected in the terminology used to denote the phenomenon(/a) – which is currently usually biased toward either a psychopathological or a political discourse, as well as in the applied measurement tools.

## Author Contributions

JA: conceptualization, data collection, data analysis, and writing – original draft. EW: conceptualization, data collection, data analysis, and writing – review and editing. All authors approved the final version of the manuscript.

## Funding

This study was supported by the Research Foundation Flanders (FWO, Belgium; grant 1152421N to JA). The University for Humanistic Studies provided funds for open access publication fees. These funding sources had no role in the study design, collection, analysis, or interpretation of the data.

## Conflict of Interest

The authors declare that the research was conducted in the absence of any commercial or financial relationships that could be construed as a potential conflict of interest.

## Publisher’s Note

All claims expressed in this article are solely those of the authors and do not necessarily represent those of their affiliated organizations, or those of the publisher, the editors and the reviewers. Any product that may be evaluated in this article, or claim that may be made by its manufacturer, is not guaranteed or endorsed by the publisher.

## Footnotes

^1^More detailed information on the methodology can be found on the OSF page of the study: https://osf.io/c48j6/.
