# Supplementary figures and images for: Older Adults Who Experience Their Lives to Be Completed and No Longer Worth Living: A Systematic Mini-Review Into Used Terminology, Definitions, and Interpretations
Source: Front Psychol. 2021 Oct 21;12:734049. doi: 10.3389/fpsyg.2021.734049 (PMC8566750; doi:10.3389/fpsyg.2021.734049)

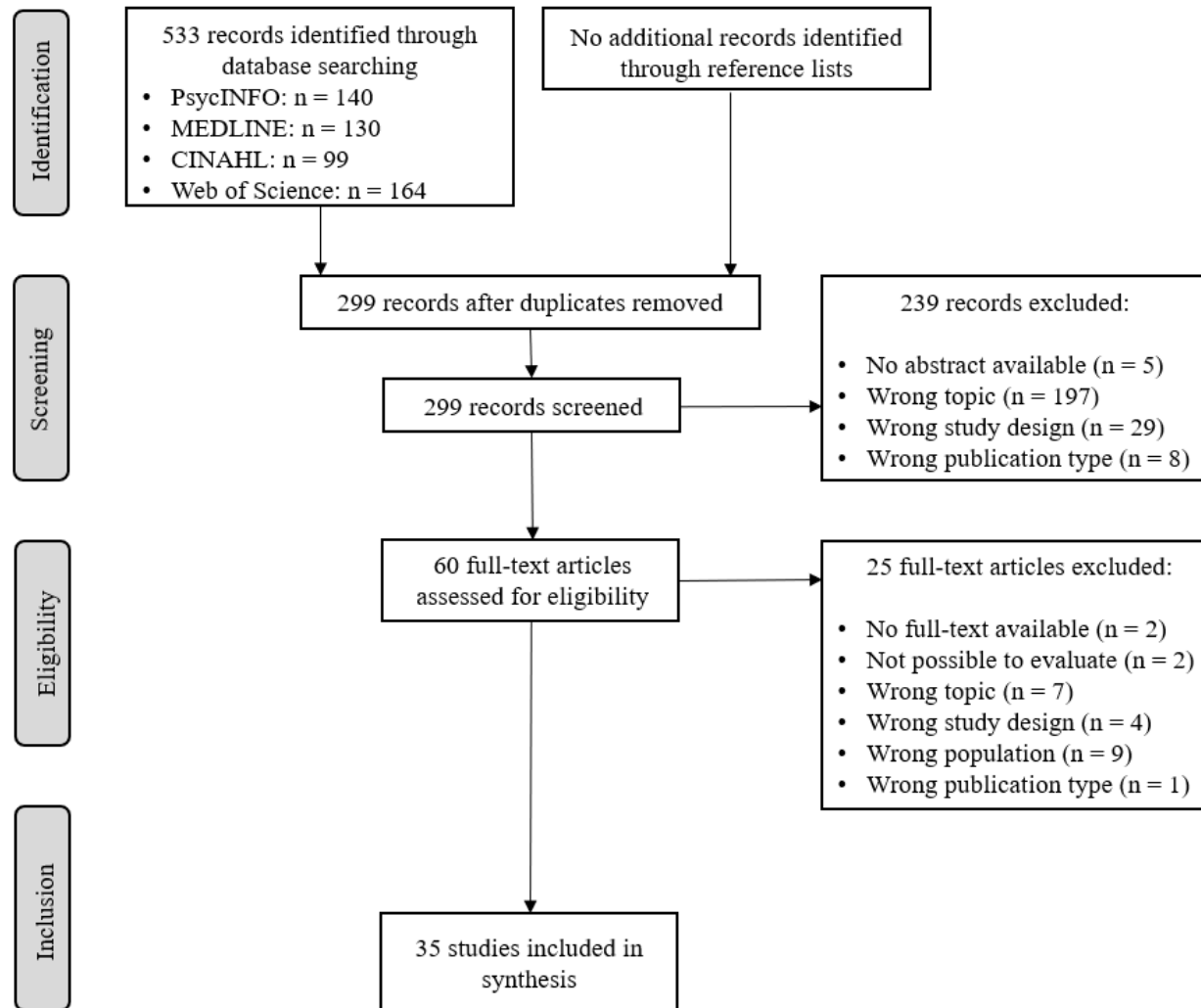

**Supplementary Figure 1.** PRISMA flow chart.

Supplement: Supplementary file 1 [file Image_1.pdf]
